# Necrotizing retinitis in a patient with syphilis: A case report

**DOI:** 10.1097/MD.0000000000024452

**Published:** 2021-03-05

**Authors:** Yan Cheng, Chenguang Wang, Guanfang Su

**Affiliations:** Eye Center of the Second Hospital, Jilin University, Changchun City, Jilin Province, China.

**Keywords:** case report, necrotizing retinitis, syphilis

## Abstract

**Rationale:**

Ocular syphilis varies widely in presentation and should be considered in all patients with posterior uveitis. Necrotizing retinitis is a rare manifestation of ocular syphilis and mimics ARN.

**Patient concerns:**

We report a male patient who presented with bilateral dense vitritis obscuring fundus details similar to ARN, as a rare reported manifestation of syphilis, who was initially given intravitreal ganciclovir.

**Diagnosis:**

After the results for herpes viral PCR disclosed negative, the diagnosis of syphilitic necrotizing retinitis was made based on positive RPR.

**Intervention and Outcomes:**

With the clinical diagnosis of ocular syphilis, treatment with intravenous penicillin was promptly initiated. His visual acuity improved to 20/100 in the right eye and still light perception in the left. Pars plana vitrectomy with silicon oil tamponade was performed in his left eye.

**Lessons:**

Ocular syphilis varies widely in presentation and should be considered in all patients with posterior uveitis. However, whenever ARN is clinically suspected, empiric treatment against herpetic viruses should be promptly administered while awaiting further infectious disease study results. Recognition of syphilitic retinitis and prompt initiation of intravenous penicillin is of critical important for clinicians.

## 1 Introduction

The etiologies of necrotizing retinitis include viral infection, such as herpes simplex and cytomegalovirus, as well as non-viral infection, including toxoplasmosis, lymphoma and syphilis. Regarding the protean ocular manifestations of syphilis, “the great imitator”, it is challenging for ophthalmologists to distinguish syphilitic retinitis from necrotizing herpetic retinitis, toxoplasmic retinochoroiditis, or lymphomatous infiltration of the retina, particularly in the setting of severe vitritis. In our report, we described a male patient who presented with bilateral severe vitritis, initially managed with empiric antiviral therapy for acute retinal necrosis (ARN), whose workup revealed ocular syphilis.

## 2 Case presentation

**Figure 1:**
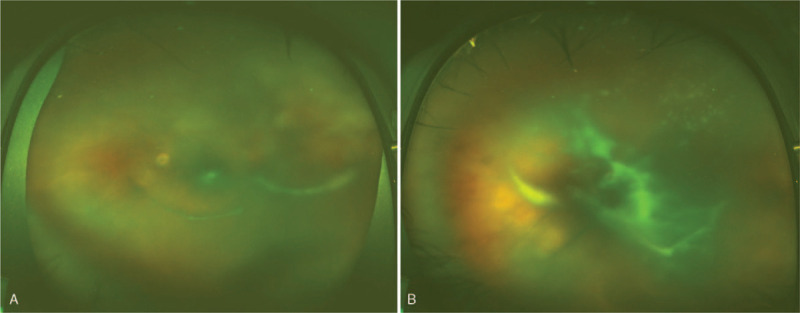
Optos photography revealed severe vitritis in the right eye (A) and in the left (B).

**Figure 2:**
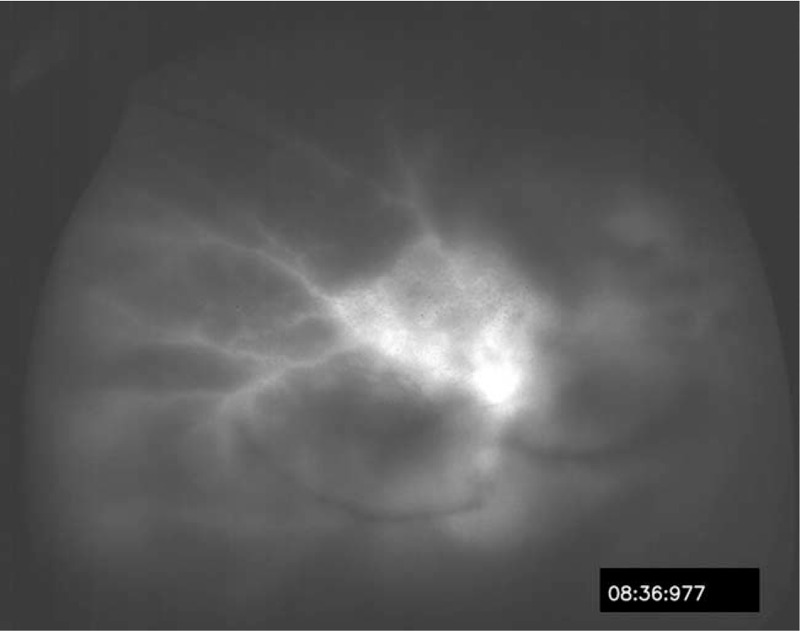
Fluorescence angiography disclosed staining of vascular wall and late leakage along posterior retinal arteriole OD.

A man in his fifties presented with a 4-week history of progressively worsening decreased vision in both eyes. His medical and surgical history included genital warts surgery. His drug history included irregular use of some medicine to increase sexual desire. He had no systemic symptoms and denied having homosexual contacts. His best-corrected visual acuity was counting figures OD and light perception OS. Extraocular movement was full and intraocular pressure was normal in both eyes. Anterior segment examinations showed moderate cell and flare in both eyes. Posterior segment examination on the right eye revealed dense vitritis and possible peripheral retinal whitening superiorly, compatible with necrotizing retinitis (Fig. 1A). A severe vitritis obscured visualization of the retina OS (Fig. 1B). Given the concern bilateral very dense vitritis suggestive of acute retinal necrosis, the patient was treated empirically with intravitreal ganciclovir without intravenous anti-viral drugs considering his slight abnormal liver function, while workup is concurrently conducted to evaluate for other possible etiologies. On the third day after admission, laboratory testing revealed positive hepatitis C virus (HCV) and positive Treponema antibody, positive serum rapid plasma reagin (RPR) at a titer of 1:32, and higher level of ALT 322 U/L (9–50) and AST 102 U/L (15–40). However, polymerase chain reaction (PCR) testing of aqueous fluid was negative for herpes simplex virus (HSV), Varicella-Zoster virus (VZV), cytomegalovirus (CMV) and Epstein-Barr (EB) DNA. Results of TORCH (toxoplasma IgM, Rubella. Virus IgM, CMV IgM, HSV IgM) and human immunodeficiency virus (HIV) were negative, and X-ray of chest was normal. Fluorescence angiography disclosed staining of vascular wall and late leakage along posterior retinal arteriole OD and a severe vitritis precluding an adequate view of the fundus OS (Fig. 2). He was diagnosed with syphilitic necrotizing retinitis. While hospitalized he refused to perform lumbar puncture.

**Figure 3:**
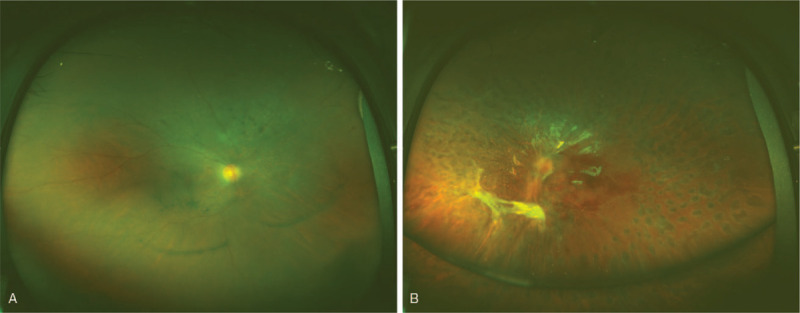
Optos photography revealed pigmentation disorder in the right eye (A) and diffuse laser dots in the left (B).

With the clinical diagnosis of ocular syphilis, aqueous crystalline penicillin G intravenous 24 million units/day was started. At the time of completion of 14 days of penicillin therapy, dramatic resolution of the vitreous opacities and retinitis was observed OD (Fig. 3A). His visual acuity improved to 20/100 in the right eye and still light perception in the left. After discharging from hospital he performed a lumbar puncture to evaluate for neurosyphilis. The results of cerebrospinal fluid revealed elevated 45 white blood cells/μl, mildly increased glucose 5.31 mmol/L and protein 0.50 g/L. RPR returned positive at a titer of 1:2. Two months later there was no significant visual improvement OS and severe vitreous opacities prevented fundus examination. Therefore pars plana vitrectomy with silicon oil tamponade was performed in his left eye (Fig. 3B). His visual acuity was hand motion in the left eye.

## 3 Discussion and conclusion

The patient was diagnosed with necrotizing retinitis due to syphilis based on the severe posterior uveitis and positive serologic testing for syphilis and negative PCR analysis for viral retinitis. Ocular syphilis varies widely in presentation and can affect both anterior and posterior segment. It should be considered in all patients with posterior uveitis. Necrotizing retinitis is a rare manifestation of ocular syphilis and mimics ARN.^[1,2]^ There are rare reported cases in ocular syphilis presenting as necrotizing retinitis. The first necrotizing retinitis with syphilis was reported by Mendelsohn.^[3]^ Shinha et al described a case of necrotizing retinitis whose fundoscopy showed vitritis and extensive white-yellow retinal lesions due to syphilis in a patient with acquired immunodeficiency syndrome (AIDS).^[4]^ We report a significant diagnostic challenging case of a patient who presented with bilateral very dense vitritis similar to ARN.

Acute retinal necrosis is often fulminant and can rapidly result in permanent vision loss. Hence, whenever acute retinal necrosis is clinically suspected, empiric treatment against herpetic viruses should be promptly administered while awaiting further infectious disease study results.^[5]^ Given the concern for ARN, the patient was initially given intravitreal ganciclovir. After the results for herpes viral PCR disclosed negative, treatment with intravenous penicillin was promptly initiated.

Syphilitic retinitis generally responds well to penicillin therapy with a greater degree of visual recovery. However, the presence of ocular symptoms for >28 days before diagnosis was significantly associated with poorer vision outcome.^[6]^ In this report, the patient presented with a long history and a more severe vision-threatening vueitis.

The challenging point in this case is that his clinical presentation mimics ARN. Recognition of syphilitic retinitis is of critical important for clinicians. We believe that this case is of great interest due to bilateral dense vitritis with no view of fundus as a rare reported manifestation of syphilis. To our best knowledge, there are no other reports in literature describing bilateral severe necrotizing retinitis in syphilis patients among children or adults.

## 4 Consent for publication

Informed written consent was obtained from the patient for publication of this case report.

## Acknowledgments

We are grateful to the patient, who gave his informed consent for publication. I would like to express my gratitude to all those who helped me during the writing of this case report.

## Author contributions

**Methodology:** Guanfang Su.

**Project administration:** Yan Cheng, Chenguang Wang.

**Resources:** Guanfang Su.

**Supervision:** Chenguang Wang, Guanfang Su.

**Writing – original draft:** Yan Cheng.

**Writing – review & editing:** Chenguang Wang, Guanfang Su.
